# Nectin-4 is frequently expressed in primary salivary gland cancer and corresponding lymph node metastases and represents an important treatment-related biomarker

**DOI:** 10.1007/s10585-023-10222-w

**Published:** 2023-07-22

**Authors:** Marcel Mayer, Lisa Nachtsheim, Johanna Prinz, Sami Shabli, Malte Suchan, Jens Peter Klußmann, Alexander Quaas, Christoph Arolt, Philipp Wolber

**Affiliations:** 1 Department of Otorhinolaryngology, Head and Neck Surgery, Medical Faculty, University of Cologne, Cologne, Germany; 2 Department I of Internal Medicine, Center for Integrated Oncology Aachen Bonn Cologne Duesseldorf, University of Cologne, Cologne, Germany; 3 Medical Faculty, Institute of Pathology, University of Cologne, Cologne, Germany

**Keywords:** Salivary gland neoplasm, Salivary duct carcinoma, Immunohistochemistry, Head and neck cancer, Targeted therapy, Nectin-4, Prognosis, Enfortumab vedotin

## Abstract

Many locally advanced and metastatic salivary gland carcinomas (SGC) lack therapeutic targets. *Enfortumab vedotin*, an antibody–drug conjugate binding to Nectin-4, recently gained FDA approval for third-line urothelial carcinoma. Therefore, the aim of this study was to assess the expression of Nectin-4 in primary SGC and corresponding lymph node metastases and to correlate it with clinicopathological data. Immunohistochemical staining for Nectin-4 was performed for patients who had undergone surgery with curative intent for primary SGC of the parotid or submandibular gland in a tertiary referral center between 1990 and 2019. One hundred twenty-two primary SGC and twenty corresponding lymph node metastases were included. Nectin-4 was expressed in 80.3% of primary SGC with a mean Histo(H-)score of 61.2 and in 90.0% of lymph node metastases with a mean H-score of 75.6. A moderate or high Nectin-4 expression was found in 25.9% of salivary duct carcinomas (SaDu) and in 30.7% of adenoid cystic carcinomas (ACC). SaDu patients with a lower T-stage (p = 0.04), no loco-regional lymph node metastases (p = 0.049), no vascular invasion (p = 0.04), and no perineural spread (p = 0.03) showed a significantly higher mean Nectin-4 H-score. There was a statistical tendency towards a more favorable disease-free survival among SaDu patients with a higher Nectin-4 expression (p = 0.09). Nectin-4 is expressed in SGC and therefore represents a potential therapeutic target, especially in entities with a high rate of local recurrence and metastatic spread such as SaDu and ACC.

## Introduction

Salivary gland carcinomas (SGC) are rare tumors constituting around 6% of all malignant tumors of the head and neck region [1]. Most SGC originate from the parotid gland, followed by the submandibular, the sublingual, and the small salivary glands [2]. SGC represent a group of more than 20 entities, each with distinct biologic and clinical characteristics [3]. For instance, while the 5-year overall survival (OS) in patients with secretory carcinoma (SeC) is 95% [4], 54% of patients with salivary duct carcinoma (SaDu) develop local recurrence/or metastatic disease resulting in an OS of around 40% [5].

To date, therapeutic options in recurrent and metastatic SGC are limited. Traditionally used platinum-based chemotherapy regimens have shown response rates of 30–40%, associated with marked toxicity [6, 7]. In recent years, molecular profiling studies have revealed several potential therapeutic targets with varying expression and response rates, e.g., the androgen receptor (AR), HER2, NTRK gene fusions, NOTCH mutations, TROP-2, and CD138 [8–12]. Although AR and HER2 are two of the most promising molecular targets, clinical trials still show limited duration of benefit with a median progression-free survival of 8.8 and 8.9 months in AR positive patients treated with androgen blockade and HER2-positive patients treated with trastuzumab-docetaxel, respectively [13, 14]. Moreover, many advanced SGC still lack therapeutic targets, leading to limited therapeutic options in the recurrent and metastatic setting. Therefore, the identification of further molecular targets is an unmet clinical need.

Nectin-4, also known as poliovirus receptor-like 4 (PVRL4), is an immunoglobulin-like transmembrane protein physiologically involved in the Ca^2+^-independent formation of adherens junctions and tight junctions between cells as well as in cell movement and survival [15, 16]. In healthy tissue, Nectin-4 is mainly expressed in embryogenic and placental cells [17, 18]. An overexpression of Nectin-4 has previously been found in bladder, breast, ovarian, pancreatic, hepatocellular, and gastrointestinal carcinomas [19–21]. Its biological role in cancerous tissue consists of activation of the PI3K/AKT pathway resulting in an overexpression of VEGF and promotion of angiogenesis [22, 23]. Further, the activation of the PI3K/AKT pathway results in an increased downstream activity of the Ras-related C3 botulinum toxin substrate 1 (Rac1) leading to cell proliferation, growth, and migration. Activated Rac1 can in turn trigger epithelial-mesenchymal transition initiating metastatic spread [21, 24].

Nectin-4 has gained significant clinical importance as a molecular target after the development of *enfortumab vedotin* (EV), an antibody–drug conjugate (ADC) consisting of a human IgG1 antibody with high affinity for Nectin-4 and monomethyl auristatin E (MMAE), a microtubule-disrupting agent. After binding to Nectin-4 expressing cells, the ADC is internalized into the tumor cell, MMAE is released, and leads to apoptosis of the cell [25]. A positive correlation between Nectin-4 expression levels and efficacy of EV was shown in vivo [26]. EV has recently been approved by the FDA for patients with advanced urothelial cancer as a third line therapy after treatment with platinum-based chemotherapy and a PD(L)-1 inhibitor [27].

To date, Nectin-4 expression in SGC has not been investigated. Therefore, the current study aimed to investigate the expression of Nectin-4 in a large SGC cohort consisting of primaries and corresponding lymph node metastases and correlating it with clinicopathological data.

## Methods

### Patient cohort and tumor characteristics

All patients with sufficient formalin-fixed paraffin-embedded (FFPE) material of the primary tumor who had undergone surgery with curative intent for primary SGC of the parotid or submandibular gland at the Department of Otorhinolaryngology, Head and Neck Surgery of the University Hospital of Cologne, Germany between 1990 and 2019 were included in this study.

Demographics, survival and histopathological data were extracted from clinical records and histopathological reports with respect to entities and stage of disease at the time of diagnosis according to the AJCC TNM staging system (8th edition, 2020) [28]. In case of missing data within the clinical records, patients or their general practitioners were phoned to follow-up on current tumor status.

The study was conducted in accordance with the Declaration of Helsinki and approved by the Ethics Committee of the University of Cologne (Approval Code: 13-091).

### Tissue microarray preparation and immunohistochemical assessment of Nectin-4 expression

Four tissue cylinders per case with a diameter of 1.2 mm per cylinder were punched out from one tumor-bearing FFPE block using a semiautomated precision instrument. The cylinders were then transferred to empty FFPE blocks to finalize the tissue microarrays (TMA). 568 tissue cylinders represented 122 cases of primary SGC and 20 lymph node metastases. A selection from the following tests to resolve unequivocal diagnoses in the cohort was used as described before [12, 29]: immunohistochemistry (IHC) staining for CK7, p63, NOR-1, SOX10, androgen receptor and HER2, FISH break-apart probes targeting *MYB, MYBL1, PRKD1, PRKD2, PRKD3, EWSR1, MAML2,* and *ETV6* genes as well as Sanger sequencing of *PRKD1* hotspot mutations [30]. Tissue slides were stained with antibodies against Nectin-4 (Abcam, clone: EPR15613-68, host: Rabbit, dilution: 1:1000, pretreatment: EDTA). All IHC stainings were carried out with a Leica BOND-MAX stainer (Leica Biosystems, Wetzlar, Germany) in accordance with the manufacturer’s protocol. Counterstaining was done using haematoxylin and bluing reagent.

Two pathologists with special expertise in the field of SGC (CA, AQ) assessed the Nectin-4 expression for each tissue cylinder on the TMAs blinded to the clinicopathological data. Cytoplasmatic and membranous staining of tumor cells was assessed as positive in accordance with previous studies evaluating Nectin-4 expression in head and neck squamous cell carcinoma (HNSCC) [31] and in urothelial carcinoma [32, 33]. Expression was assessed using the semi-quantitative Histo-(H-)score [34], which consists of the product of the staining intensity (0–3) and the percentage of cells stained at each intensity level (0–100). Thus, the H-score ranges between 0 (0% cells stained) and 300 (100% * 3). The final H-score represents the mean value of the four cylinders per case. Finally, cases were classified as negative (H-score = 0), low (H-score 1–100), moderate (101–200), and high (201–300).

### Statistical analysis

Statistical analyses were performed using SPSS software version 28.0.0.0 (190) (IBM, Armonk, NY). Distribution was tested using the Shapiro–Wilk test. The Mann–Whitney-*U* test was used to compare differences between two independent groups for metric, non-normally distributed variables. The Wilcoxon signed-rank test was used to compare differences between two matched pairs for metric, non-normally distributed data. The Kaplan–Meier method with 95% confidence intervals was used to test for disease-free survival (DFS) probability rates. For the survival analysis, patients were dichotomized in subgroups with Nectin-4 H-scores of ≥ 100 and < 100. In this context, the log-rank test was used for testing for statistical significance. DFS was defined as the time interval between the end of treatment and the date of recurrence or death. A p-value < 0.05 was considered statistically significant. R studio (version 2021.09.1) was used for visualization of box plots (ggplot2 package).

## Results

### Patients’ cohort

One hundred twenty-two patients with primary SGC of the parotid (91.0%) and submandibular gland (9.0%) were included. The most frequent entities were salivary duct carcinoma (SaDu; 22.1%, n = 27), adenoid cystic carcinoma (ACC; 21.3%, n = 26), and mucoepidermoid carcinoma (MuEp; 20.5%, n = 25), followed by acinic cell carcinoma (Acin; 10.7%, n = 13), epithelial-myoepithelial carcinoma (EpMy; 7.4%, n = 9), and secretory carcinoma (SeC; 5.7%, n = 7). Other rare entities (OTH; 12.3%, n = 15) were four basal cell carcinomas (3.3%), four adenocarcinomas not otherwise specified (ANOS; 3.3%), three myoepithelial carcinomas (2.5%), one oncocytic cell carcinoma (0.8%), one carcinosarcoma (0.8%), one polymorphous adenocarcinoma (PAC; 0.8%), and one poorly differentiated carcinoma (0.8%). For the whole cohort, gender distribution was equal (females: 51.6%, males: 48.4%). Most patients with SaDu were male (77.8%) and most patients with MuEp (72.0%) were female. Mean age across all patients was 56.4 years. Fifty-nine patients (48.4%) had an advanced pathological T-stage 3/4. Thirty-eight patients (31.1%) showed locoregional lymph node metastases. Further demographic and histopathological data are presented in Table 1.

**Table 1:** Localization of the primary tumor, demographic data, histopathological data, mean Nectin-4 H-Score, and Nectin-4 expression grouped in high (H-Score 200–300), moderate (H-Score 100–199), low (H-Score 1–99), negative (H-Score = 0) for the most frequent entities

	All n = 122	SaDu n = 27	ACC n = 26	MuEp n = 25	Acin n = 13	EpMy n = 9	SecC n = 7	OTH n = 15
Localization								
Parotid gland	111 (91.0)	25 (92.6)	19 (73.1)	25 (100.0)	13 (100.0)	9 (100.0)	6 (85.7)	14 (93.3)
Submandibular gland	11 (9.0)	2 (7.4)	7 (26.9)	0 (0.0)	0 (0.0)	0 (0.0)	1(14.3)	1 (6.7)
Demographics								
Female	63 (51.6)	6 (22.2)	16 (61.5)	18 (72.0)	8 (61.5)	3 (33.3)	3 (42.99)	9 (60.0)
Male	59 (48.4)	21 (77.8)	10 (38.5)	7 (28.0)	5 (38.5)	6 (66.7)	4 (57.1)	6 (40.0)
Age	56.4 ± 17.7	67.7 ± 11.5	50.9 ± 13.4	44.6 ± 18.3	55.0 ± 19.3	63.9 ± 17.3	48.6 ± 20.0	66.1 ± 10.4
Histopathological parameters T classification								
T1-2	59 (48.4)	10 (37.0)	11 (42.3)	17 (68.0)	6 (46.2)	6 (66.7)	5 (71.4)	4 (26.7)
T3-4	59 (48.4)	17 (63.0)	14 (53.8)	7 (28.0)	7 (53.8)	3 (33.3)	2 (28.6)	9 (60.0)
N/A	4 (3.2)	0 (0.0)	1 (3.8)	1 (4.0)	0 (0.0)	0 (0.0)	0 (0.0)	2 (13.3)
N classification								
N0	80 (65.6)	4 (14.8)	18 (69.2)	21 (84.0)	10 (76.9)	9 (100.0)	6 (85.7)	12 (80.0)
N+	38 (31.1)	23 (85.2)	7 (26.9)	3 (12.0)	2 (15.4)	0 (0.0)	1 (14.3)	2 (13.3)
N/A	4 (3.3)	0 (0.0)	1 (3.8)	1 (4.0)	1 (7.7)	0 (0.0)	0 (0.0)	1 (6.7)
Vascular invasion								
V0	99 (81.1)	21 (77.8)	20 (76.9)	23 (92.0)	12 (92.3)	8 (88.9)	6 (85.7)	9 (60.0)
V1	12 (9.8)	5 (18.5)	1 (3.8)	2 (8.0)	0 (0.0)	1 (11.1)	1 (14.3)	3 (20.0)
N/A	11 (9.1)	1 (3.7)	5 (19.2)	0 (0.0)	1 (7.7)	0 (0.0)	0 (0.0)	3 (20.0)
Perineural invasion								
Pn0	68 (55.7)	5 (18.5)	10 (38.5)	22 (88.0)	10 (76.9)	8 (88.9)	6 (85.7)	7 (46.7)
Pn1	44 (36.1)	22 (81.5)	12 (46.2)	2 (8.0)	2 (15.4)	0 (0.0)	1 (14.3)	5 (33.3)
N/A	10 (8.2)	0 (0.0)	4 (15.4)	1 (4.0)	1 (7.7)	1 (11.1)	0 (0.0)	3 (20.0)
Lymphovascular invasion								
L0	95 (77.9)	15 (55.6)	20 (76.9)	24 (96.0)	10 (76.9)	8 (88.9)	7 (100.0)	11 (73.3)
L1	17 (13.9)	11 (40.7)	1 (3.8)	1 (4.0)	2 (15.4)	1 (11.1)	0 (0.0)	1 (6.7)
N/A	10 (8.2)	1 (3.7)	5 (19.2)	0 (0.0)	1 (7.7)	0 (0.0)	0 (0.0)	3 (20.0)
Extracapsular extension								
ECE−	94 (77.0)	14 (51.9)	18 (69.2)	24 (96.0)	11 (84.6)	9 (100.0)	7 (100.0)	11 (73.3)
ECE+	19 (15.6)	13 (48.1)	3 (11.5)	1 (4.0)	1 (7.7)	0 (0.0)	0 (0.0)	1 (6.7)
N/A	9 (7.4)	0 (0.0)	5 (19.2)	0 (0.0)	1 (7.7)	0 (0.0)	0 (0.0)	3 (20.0)
Grading								
Low	27 (22.1)	7 (25.9)	1 (3.8)	15 (60.0)	1 (7.7)	0 (0.0)	2 (28.6)	1 (6.7)
High/intermediate	45 (45.1)	17 (63.0)	18 (69.2)	10 (40.0)	2 (15.4)	1 (11.1)	1 (14.3)	6 (40.0)
N/A	40 (32.8)	3 (11.1)	7 (26.9)	0 (0.0)	10 (76.9)	8 (88.9)	4 (57.1)	8 (53.3)
Nectin-4 (Mean H-Score; ±)	61.2 ± 65.7	78.3 ± 67.0	68.6 ± 75.6	58.1 ± 59.9	65.4 ± 90.6	78.5 ± 48.2	35.4 ± 34.5	20.9 ± 27.8
Nectin-4 expression								
High	6 (4.9)	2 (7.4)	1 (3.8)	1 (4.0)	2 (15.4)	0 (0.0)	0 (0.0)	0 (0.0)
Moderate	18 (14.8)	5 (18.5)	7 (26.9)	3 (12.0)	0 (0.0)	3 (33.3)	0 (0.0)	0 (0.0)
Low	74 (60.7)	16 (59.3)	13 (50.0)	17 (68.0)	8 (61.5)	6 (66.7)	7 (100.0)	7 (46.7)
Negative	24 (19.7)	4 (14.8)	5 (19.2)	4 (16.0)	3 (23.1)	0 (0.0)	0 (0.0)	8 (53.3)

*n* number of patients, () percentages, ± standard deviation, *MuEp* Mucoepidermoid carcinoma, *EpMy* epithelial-myoepithelial carcinoma, *Acin* acinic cell carcinoma, *SaDu* salivary duct carcinoma, *ACC* adenoid cystic carcinoma, *SeC* secretory carcinoma, *OTH* others

### Immunohistochemistry

**Fig. 1:**
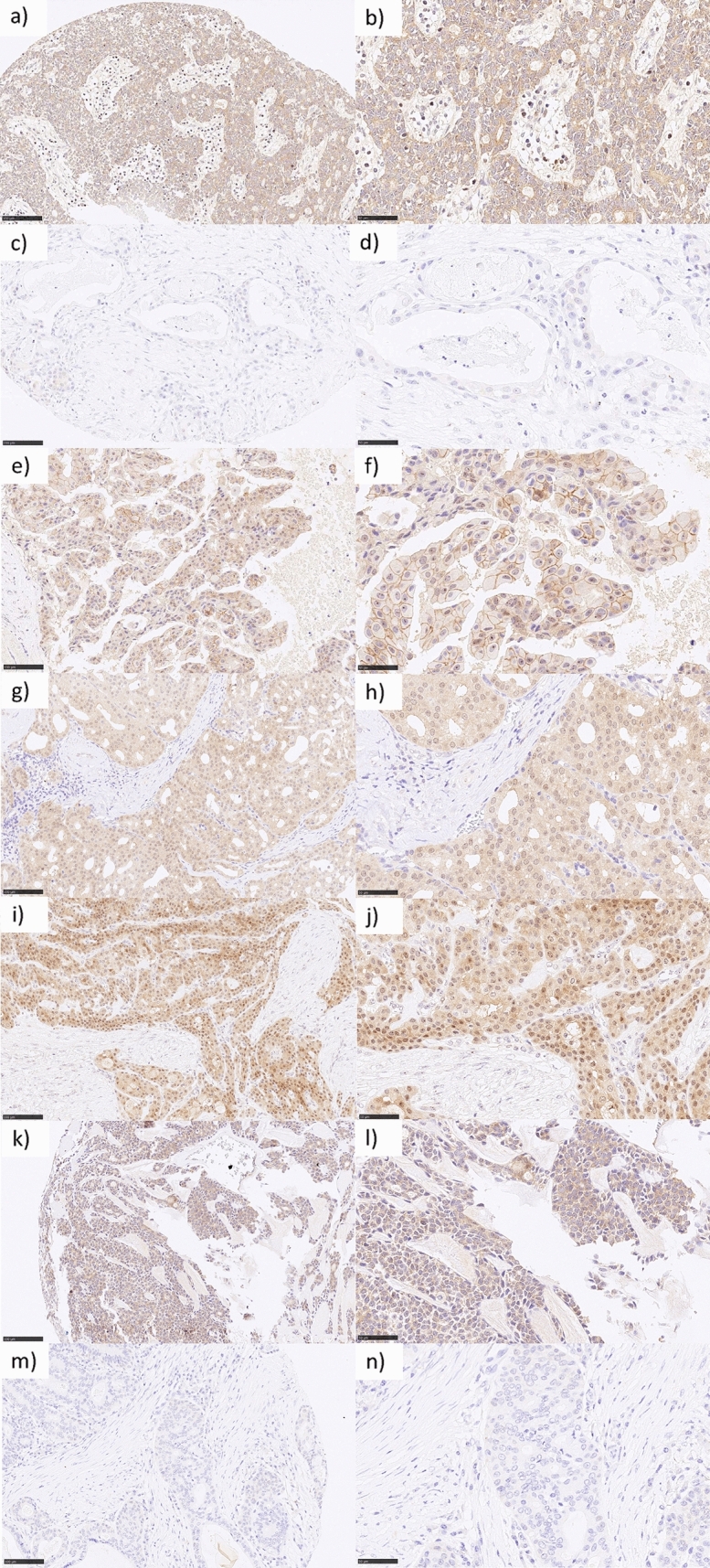
Nectin-4 immunohistochemistry in salivary gland carcinomas: **a** (200×) and **b** (400×) adenoid cystic carcinoma with moderate cytoplasmatic staining in 100% of tumor cells accounting for an H-score of 200. **c** (200×) and **d** (400×) mucoepidermoid carcinoma with negativity for Nectin-4. **e** (200×) and **f** (400×) salivary duct carcinoma with mixed moderate membranous and cytoplasmatic staining in 100% of tumor cells accounting for an H-score of 200. **g** (200×) and **h** (400×) acinic cell carcinoma with high cytoplasmatic staining in 100% of tumor cells accounting for an H-score of 300. **i** (200×) and **j** (400×) mucoepidermoid carcinoma with high cytoplasmatic staining in 90% of tumor cells accounting for an H-score of 270. **k** (200×) and **l** (400×) epithelial-myoepithelial carcinoma with moderate cytoplasmatic staining in 90% of tumor cells accounting for an H-score of 180. **m** (200×) and **n** (400×) salivary duct carcinoma with negativity for Nectin-4

**Fig. 2:**
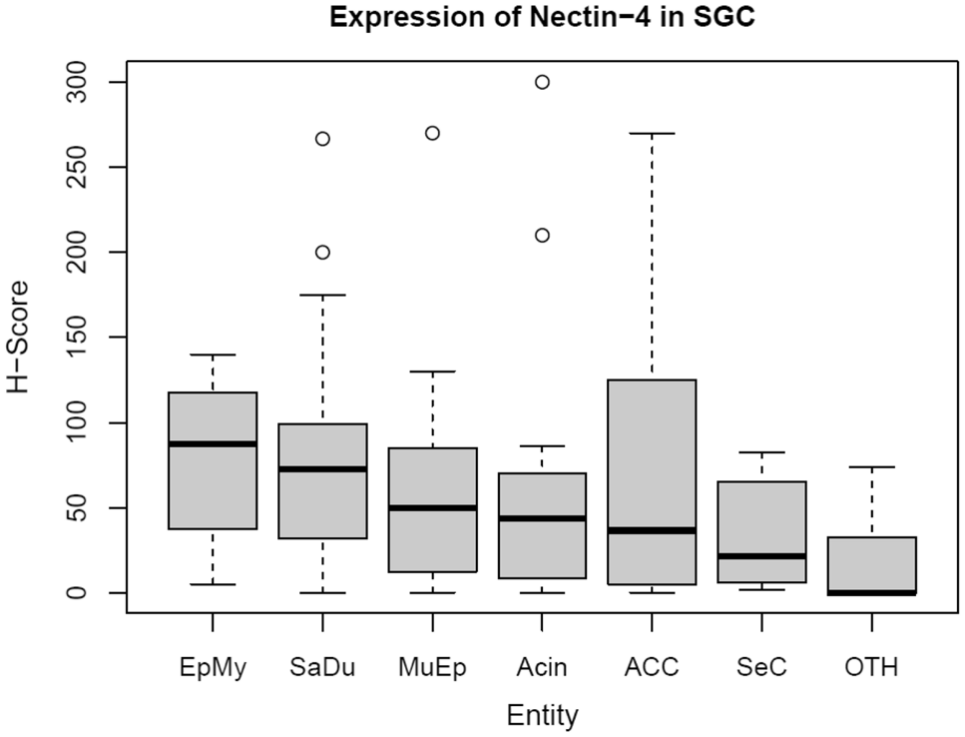
Box plot displaying the distribution of Nectin-4 expression among the most frequent entities. *SGC* salivary gland cancer, *MuEp* mucoepidermoid carcinoma, *EpMy* epithelial-myoepithelial carcinoma, *Acin* acinic cell carcinoma, *SaDu* salivary duct carcinoma, *ACC* adenoid cystic carcinoma, *SeC* secretory carcinoma, *OTH* others. *H-score* histoscore. *Max. H-score* 300

**Fig. 3:**
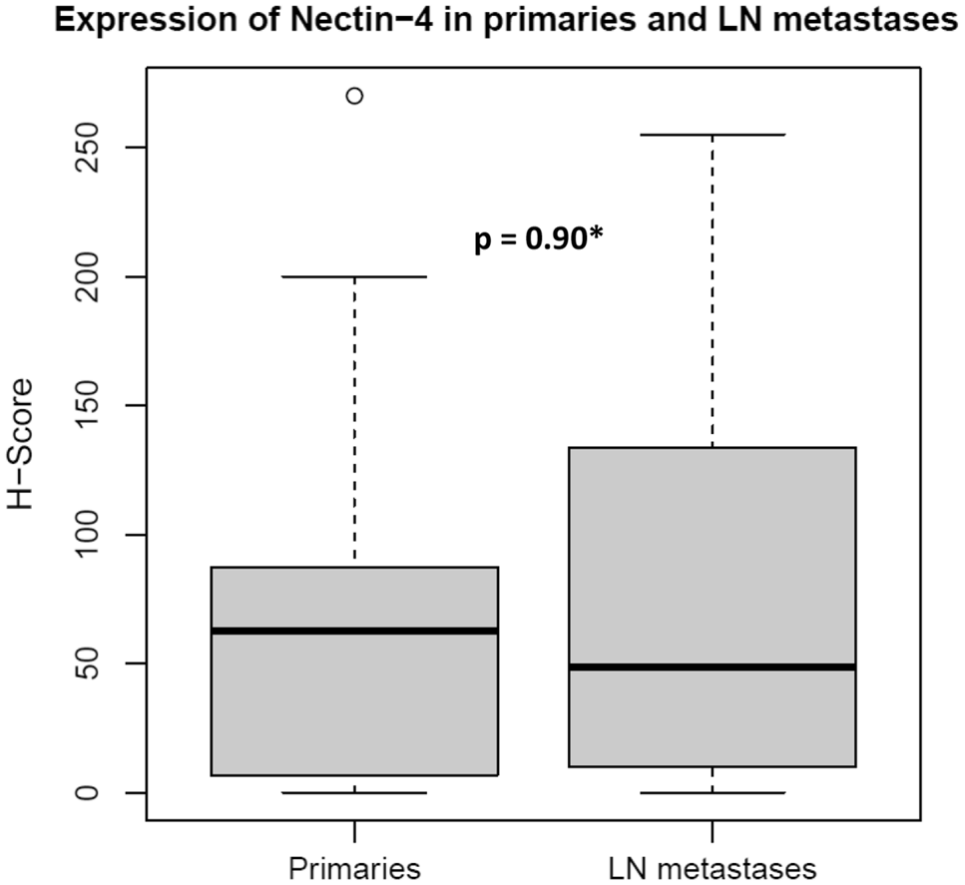
Box plot displaying the Nectin-4 expression among primary and lymph node metastatic salivary gland cancer. *LN metastases* lymph node metastases. *Wilcoxon rank-sum test. *H-score* histoscore. *Max. H-score* 300

The mean Nectin-4 H-score was 61.2 (± 65.7) among all primaries. The highest expression was found in EpMy (mean H-Score: 78.5 ± 48.2) and SaDu (78.3 ± 67.0), followed by ACC (68.6 ± 75.6), Acin (65.4 ± 90.6), MuEp (58.1 ± 59.9), and SeC (35.4 ± 34.5). Overall, 80.3% of the cases showed a Nectin-4 expression. Among these, a moderate or high expression was found in 24.5%, whereas a low expression was found in 75.5% of cases (Table 1). Figure 1 shows the immunohistochemical protein expression of Nectin-4 for exemplary cases. Figure 2 displays a box plot of the expression of Nectin-4 for the most frequent entities. Among 20 included loco-regional lymph node metastases, the mean Nectin-4 H-score was 75.6 (± 77.9). Nectin-4 was expressed in 18 out of 20 (90.0%) of those. The mean Nectin-4 H-scores in the lymph node metastases were as follows: MuEp 157.5% (n = 1), ACC 144.6% (n = 3, ± 87.5), SaDu 68.9% (n = 11, ± 80.4), Acin 44.6% (n = 3, ± 42.0), 16.3% (n = 1), and ANOS 12.5% (n = 1). Nectin-4 expression among primaries and loco-regional lymph node metastases did not differ (p = 0.90). The Nectin-4 expression for primaries and loco-regional lymph node metastases is displayed in Fig. 3. Membranous Nectin-4 expression was seen in 30.3% of primaries and 55.0% of lymph node metastases, whereas cytoplasmatic expression was seen in 77.0% of primaries and 80.0% of lymph node metastases, respectively. A convincing nuclear expression across the included primaries or lymph node metastases was not observed. The mean standard deviation of the H-score across the four TMAs per case was 14.80% for primaries and 13.02% for lymph node metastases. Generally, a relatively homogenous expression pattern was seen.

**Fig. 4:**
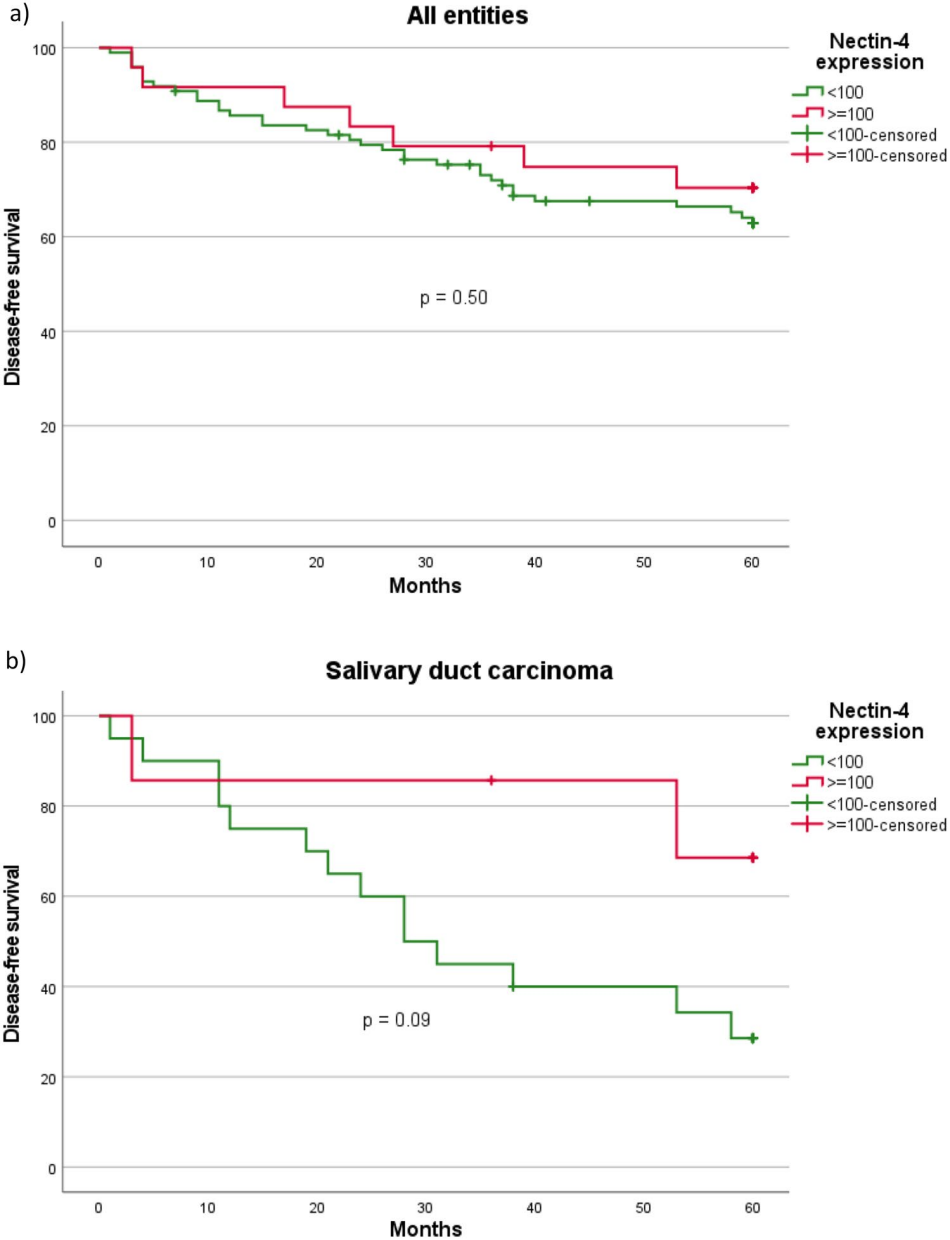
Kaplan–Meier curves and p-values of log-rank tests for **a** Nectin-4 H-Score (≥ 100 vs. < 100) among all entities, **b** Nectin-4 H-Score (≥ 100 vs. < 100) among salivary duct carcinoma. All entities = Salivary gland cancer cohort (n = 122). Salivary duct carcinoma cohort (n = 27)

The statistical association between the mean Nectin-4 H-score and localization of the primary, demographic data as well as histopathological data is displayed in Table 2. There was no significant association between localization, demographic, and histopathological variables and Nectin-4 expression for the whole cohort, MuEp, and ACC. SaDu patients with a higher T-stage (p = 0.04), loco-regional lymph node metastases (p = 0.049), vascular invasion (p = 0.04), and perineural spread (p = 0.03) showed a lower mean Nectin-4 H-score.

**Table 2:** Statistical association between localization of the primary tumor, age, sex, histopathological data and Nectin-4 expression for all entities and for the most frequent entities salivary duct carcinoma (SaDu), mucoepidermoid carcinoma (MuEp), and adenoid cystic carcinoma (ACC)

Variable	Mean Nectin-4 H-Score (all entities)	p	Mean Nectin-4 H-Score (SaDu)	p	Mean Nectin-4 H-Score (MuEp)	p	Mean Nectin-4 H-Score (ACC)	p
Parotid gland Submandibular gland	63.3 39.8	0.12^#^	81.7 36.3	0.37^#^	58.1		75.8 49.2	0.31^#^
< 60 years > = 60 years	63.5 57.7	0.60^#^	94.9 73.5	0.56^#^	63.6 38.1	0.24^#^	75.8 44.6	0.57^#^
Male Female	57.9 64.3	0.48^#^	76.5 84.6	0.84^#^	37.7 66.0	0.33^#^	63.2 72.0	0.78^#^
T1/2 T3/4	64.4 59.8	0.41^#^	113.2 57.8	**0.04**^**#**^	53.9 69.5	0.95^#^	46.9 90.2	0.23^#^
N0 N+	56.7 74.8	0.08^#^	133.9 68.6	**0.049**^**#**^	56.3 72.9	0.40^#^	56.5 97.4	0.20^#^
V0 V1	61.1 53.9	0.42^#^	92.1 36.6	**0.04**^**#**^	58.3 55.6	0.89^#^	50.0 270.0	0.10^#^
Pn0 Pn1	55.1 70.9	0.17^#^	137.1 64.9	**0.03**^**#**^	57.5 56.9	0.78^#^	93.1 65.0	0.11^#^
L0 L1	56.2 80.2	0.12^#^	81.9 77.1	1.00^#^	58.9 38.8	0.10^#^	50.0 270.0	0.10^#^
ECE− ECE+	58.0 68.2	0.44^#^	86.0 70.0	0.49^#^	58.9 38.8	0.10^#^	52.6 67.6	0.67^#^
G1 G2/3	57.3 70.0	0.57^#^	83.9 81.0	0.85^#^	44.2 78.9	0.22^#^	187.5 74.6	0.21^#^

Significance level p < 0.05, ^#^Mann–Whitney-*U* test, significant values in bold letters

### Survival

The 5-year disease-free survival (DFS) among all entities was 65.6% (80 out of 122) with a mean follow-up of 66.3 (± 52.2) months. The highest DFS was found for SeC (100.0%) and the lowest DFS for SaDu (40.7%). Among all SGC, patients with a moderate or high (≥ 100) and a negative or low (< 100) Nectin-4 H-score had a DFS of 70.8%, and 64.3% (p = 0.50), respectively. SaDu patients with a moderate or high H-score showed a DFS of 71.4%, whereas those with a negative or low H-score showed a DFS of 30.0% (Fig. 4). Although not statistically significant (p = 0.09), this represented a marked trend towards a more favorable DFS among those with a higher H-score. Among the other subgroups, DFS did not differ significantly between patients with a Nectin-4 H-score ≥ 100 and < 100 (ACC: p = 0.76; MuEp: p = 0.62; Acin: p = 0.39; EpMy: p = 0.16).

## Discussion

To date, this study is the first to evaluate the expression of Nectin-4 in SGC. More precisely, the present study aimed at assessing the Nectin-4 expression in FFPE material obtained from a large cohort of patients with different SGC entities and investigating the association between Nectin-4 expression and clinicopathological data.

Overall, 80.3% of the cases were positive for Nectin-4 with a mean H-score of 61.2 (± 65.7). Out of all cases, 19.7% had a moderate or high (H-score: 101–300) Nectin-4 expression. A previous immunohistochemical study showed a wide expression of Nectin-4 in different solid carcinomas ranging from Nectin-4 positivity in 55% of esophageal to 83% of bladder cancer with a moderate or high expression ranging from 18% in ovarian cancer to 60% in bladder cancer [26]. Two studies have assessed Nectin-4 expression in HNSCC showing positivity in 59 and 86.2% of cases with a moderate or high expression in 18 and 33.2% of cases [26, 31]. Thus, Nectin-4 expression in head and neck cancers seems to be similarly high in HNSCC and SGC.

Nectin-4 is targeted by the first-in-class ADC *enfortumab vedotin* (EV), which has been introduced in 2016. EV has shown dose-dependent inhibition of cell viability in human, rat, and monkey cell lines transfected to express Nectin-4 as well as breast cancer cell lines endogenously expressing Nectin-4. Further, EV led to a significant reduction of tumor volume in Nectin-4 positive bladder cancer, breast cancer, and lung cancer xenografts and to a significant inhibition of tumor growth in Nectin-4 positive pancreatic cancer xenografts compared to controls. There were no apparent signs of side effects in the xenografts treated with EV. A correlation between levels of Nectin-4 expression and in vivo efficacy was observed [26]. In 2019 accelerated FDA approval has been granted to EV as third-line therapy for patients with locally advanced/metastatic urothelial cancer [35]. In 2021 Powles et al. showed in a large prospective, randomized phase III study among patients with locally advanced or metastatic urothelial cancer after platinum-based chemotherapy and PD-(L)1 inhibition a significantly longer OS and progression-free survival in patients treated with EV compared to investigator-chosen chemotherapy. Adverse events in the EV group were similar, but less frequent, than in the chemotherapy group with the most frequent grade 3 events being rash, neutropenia, and fatigue [36]. Following this study, full FDA and EMA approval was granted to EV for patients with urothelial cancer who had previously received PD-(L)1 inhibition and platinum-based chemotherapy [35, 37]. To date, there is neither a study displaying the Nectin-4 expression in SGC, nor a preclinical or clinical trial that has investigated the efficacy of EV in SGC.

In this study Nectin-4 expression in SaDu and ACC was moderate or high in 25.9 and 30.7% of cases, respectively. SaDu is one of the most aggressive entities among SGC. More than 60% of patients present with lymph node metastases at first diagnosis [38] and 54% of patients develop locoregional or distant recurrences resulting in a OS of around 40% [5, 39]. Although the current European guidelines for treatment of SGC recommend the use of antiandrogen therapy and/or HER2 targeted therapy in case of AR/HER positivity [40], progression free survival in these patients has been shown to be shorter than 9 months [13, 14]. Therefore, more effective therapeutic options are needed, and EV may be a potential drug for patients with advanced SaDu with Nectin-4 expression. While ACC has a more favorable 5-year OS of around 80%, almost 75% of patients develop distant metastases in the long-term resulting in a 15-year OS of less than 30% [41]. As ACC currently lacks established therapeutic targets, a moderate or high Nectin-4 expression in almost one-third of ACC seems promising for a targeted treatment with e.g. EV.

When evaluating a potential therapeutic target, its expression in metastatic lesions is of particular importance as these lesions are most likely the ones targeted in the advanced situation. As a sufficient number of histological specimens from distant metastases in the rare entity of SGC was lacking, the Nectin-4 expression in 20 loco-regional lymph node metastases was assessed in this study. Ninety percent of these were positive for Nectin-4 as well with a mean H-score of 75.6 (± 77.9). There was no significant difference between the Nectin-4 expression in the primary tumors and corresponding lymph node metastases. Consequently, it seems that there is no loss or gain of Nectin-4 expression during lymphatic spread in SGC. Further studies are required to assess the Nectin-4 expression in distant metastatic SGC.

As shown in a previous study evaluating the efficacy of trastuzumab emtansine in advanced gastric/gastroesophageal junction cancer, patients with homogenous HER2 staining pattern had a more favorable median OS compared to those with heterogenous and focal HER2 expression [42]. Therefore, the homogeneity of expression of a potential molecular target within the tumor seems to play a role when targeted by an antibody–drug conjugate. In the present study, a homogenous expression pattern of Nectin-4 was observed in the primaries as well as in corresponding lymph node metastases. This is objectified by a mean H-score standard deviation of 14.80% across the four TMAs per case in the primaries and 13.02% in the lymph node metastases.

The role of Nectin-4 as a prognostic marker is controversial. While a higher protein expression has been shown to be associated with a worse OS in several solid tumors [21], recent studies among patients with triple-negative breast cancer and HNSCC showed a more favorable OS for tumors with a high Nectin-4 expression [31, 43]. In the present study, there was a trend towards a more favorable DFS among SaDu patients with high compared to those with low Nectin-4 expression. The most likely explanation for the lack of statistical significance is the small size of the SaDu subgroup. Nevertheless, as SaDu and ductal carcinoma of the breast have similar biological and histopathological features [44], it seems plausible that a higher Nectin-4 expression in SaDu may serve as a favorable prognostic factor. A possible mechanistic explanation for a worse prognosis in patients with low expression of Nectin-4 is a loss of adhesion between cells with an increase of cell migration after Nectin-4 knockdown, as seen in vitro in cutaneous squamous cell carcinoma cells [45]. An association between the expression of a targetable receptor and a more favorable OS has previously been shown for the AR in SaDu [46] and therefore does not hinder Nectin-4 to be a potential therapeutic target in SGC.

These results are in line with the correlation analysis for Nectin-4 expression and clinicopathological data in this study. Although there was no significant association between Nectin-4 expression and clinicopathological data for the whole cohort and for the entities MuEp, and ACC, a higher Nectin-4 expression was associated with a lower T-stage, absence of lymph node metastases, and absence of vascular or perineural invasion in SaDu. This is in accordance with an immunohistochemical study among patients with triple-negative breast cancer, where a higher Nectin-4 expression was significantly associated with a lower T-stage and absence of lymph node metastases [43].

This study has the limitations of a retrospective acquisition of clinicopathological data and the limited number of cases per entity potentially leading to insignificant results and selection bias. On the other hand, this is the first study assessing the Nectin-4 expression in SGC and correlating the expression with a large set of clinicopathological data. Also, it must be considered that the limited number of cases per entity is due to the low incidence of SGC in general, and the high number of different entities of SGC in particular.

As a conclusion, the results of the present study show that Nectin-4 is expressed in primary and metastatic SGC and that a proportion of patients with the prognostically unfavorable entities salivary duct carcinoma and adenoid cystic carcinoma show a moderate or high expression. Additionally, Nectin-4 expression may represent a prognostic factor in salivary duct carcinoma. The current results warrant the investigation of the efficacy of EV in SGC.

## Data Availability

The datasets generated and analysed during the current study are available from the corresponding author on reasonable request.
